# 2,3-Dibromo-1-(2,4-dichloro-5-fluoro­phen­yl)-3-phenyl­propan-1-one

**DOI:** 10.1107/S1600536808013238

**Published:** 2008-05-10

**Authors:** Hoong-Kun Fun, Samuel Robinson Jebas, Ibrahim Abdul Razak, M. S. Karthikeyan, P. S. Patil, S. M. Dharmaprakash

**Affiliations:** a X-ray Crystallography Unit, School of Physics, Universiti Sains Malaysia, 11800 USM, Penang, Malaysia; b Syngene International Pvt Limited, Plot No. 2 & 3 C, Unit-II, Bommansandra Industrial Area, Banglore 560 099, India; c Department of Studies in Physics, Mangalore University, Mangalagangotri, Mangalore 574 199, India

## Abstract

In the title compound, C_15_H_9_Br_2_Cl_2_FO, the dihedral angle between the two aromatic rings is 6.0 (1)°. The dibromo­ethane fragment of the propan-1-one unit is disordered over two positions, with occupancies of *ca* 0.83 and 0.17. The crystal structure is stabilized by inter­molecular C—H⋯O hydrogen bonds, C—H⋯π inter­actions, and Br⋯Cl [3.505 (2) and 3.576 (6) Å] and Cl⋯F [3.176 (2) Å] short contacts.

## Related literature

For related literature, see: Agrinskaya *et al.* (1999 ▶); Patil *et al.* (2006 ▶); John Kiran *et al.* (2007 ▶). For bond-length data, see: Allen *et al.* (1987 ▶). For the preparation, see: Shivarama Holla *et al.* 2006 ▶).
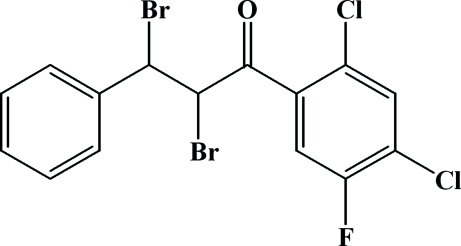


## Experimental

### 

#### Crystal data

C_15_H_9_Br_2_Cl_2_FO
*M* *_r_* = 454.94
Orthorhombic, 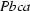

*a* = 7.1232 (1) Å
*b* = 10.0757 (2) Å
*c* = 43.0262 (7) Å
*V* = 3088.04 (9) Å^3^
*Z* = 8
Mo *K*α radiation
μ = 5.60 mm^−1^
*T* = 100 (2) K
0.40 × 0.24 × 0.14 mm

#### Data collection

Bruker SMART APEXII CCD area-detector diffractometer
Absorption correction: multi-scan (*SADABS*; Bruker, 2005 ▶) *T*_min_ = 0.211, *T*_max_ = 0.508 (expected range = 0.190–0.457)
26343 measured reflections
5857 independent reflections
3681 reflections with *I* > 2σ(*I*)
*R*_int_ = 0.056

#### Refinement

*R*[*F* ^2^ > 2σ(*F* ^2^)] = 0.041
*wR*(*F* ^2^) = 0.089
*S* = 0.98
5857 reflections
227 parameters
60 restraints
H-atom parameters constrained
Δρ_max_ = 0.77 e Å^−3^
Δρ_min_ = −0.76 e Å^−3^

### 

Data collection: *APEX2* (Bruker, 2005 ▶); cell refinement: *APEX2*; data reduction: *SAINT* (Bruker, 2005 ▶); program(s) used to solve structure: *SHELXTL* (Sheldrick, 2008 ▶); program(s) used to refine structure: *SHELXTL*; molecular graphics: *SHELXTL*; software used to prepare material for publication: *SHELXTL* and *PLATON* (Spek, 2003 ▶).

## Supplementary Material

Crystal structure: contains datablocks global, I. DOI: 10.1107/S1600536808013238/ci2588sup1.cif

Structure factors: contains datablocks I. DOI: 10.1107/S1600536808013238/ci2588Isup2.hkl

Additional supplementary materials:  crystallographic information; 3D view; checkCIF report

## Figures and Tables

**Table 1:** Hydrogen-bond geometry (Å, °)

*D*—H⋯*A*	*D*—H	H⋯*A*	*D*⋯*A*	*D*—H⋯*A*
C5—H5⋯O1^i^	0.95	2.45	3.392 (4)	170
C8—H8⋯O1^i^	1.00	2.35	3.336 (4)	169
C11—H11⋯O1^i^	0.95	2.29	3.229 (3)	170
C3—H3⋯*Cg*1^ii^	0.95	2.96	3.652 (3)	131

Symmetry codes: (i) 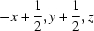; (ii) 
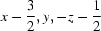
. *Cg*1 is the centroid of the C1–C6 benzene ring.

**Fig. 1.**
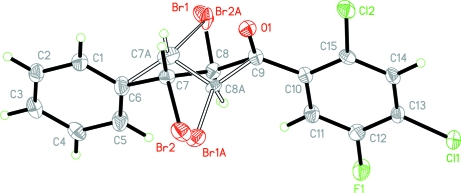
The molecular structure of the title compound, showing 50% probability displacement ellipsoids and the atom-numbering scheme. Only the major disorder component is shown.

**Fig. 2.**
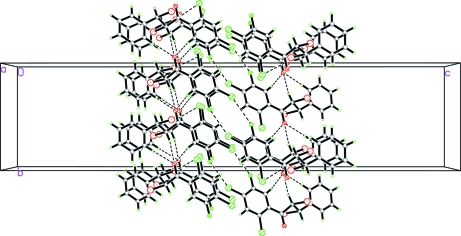
The crystal packing of the title compound, viewed along the a axis. Short intra- and intermolecular contacts and hydrogen bonds are shown as dashed lines. Only the major disorder component is shown.

## supplementary crystallographic information

### Comment

Derivatives of chalcone exhibit nonlinear optical (NLO) properties (Agrinskaya *et al.*, 1999; Patil *et al.*, 2006; John Kiran *et al.*, 2007). We report here the crystal structure of the title compound (Fig 1), which crystallizes in a centrosymmetric space group and this precludes the presence of second-order NLO properties.

Bond lengths and angles in the title molecule have normal values (Allen *et al.*, 1987). The dihedral angle between the benzene rings is 6.0 (1)°. The dibromoethane fragment of the propan-1-one unit is diordered over two positions.

The crystal packing is (Fig.2) stabilized by intermolecular C—H···O and weak C—H···π interactions involving the C1–C6 benzene ring (centroid Cg1). In addition, Br2···Cl2(-1+x,y,z) [3.505 (2) Å], Br1A···Cl2(-1+x,y,z) [3.576 (6) Å] and Cl1···F1(1-x,1-y,-z) [3.176 (2) Å] short contacts are oberved in the crystal structure.

### Experimental

1-(2,4-Dichloro-5-fluorophenyl)-3-phenylprop-2-en-1-one (1 mmol) was prepared by a literature procedure (Shivarama Holla *et al.*, 2006). To a solution of 1-(2,4-dichloro-5-fluorophenyl)-3-phenylprop-2-en-1-one) (1 mmol) in chloroform (25 ml), bromine (1 mmol) was added slowly with stirring. After the completion of addition the reaction mixture was stirred for 24 h. Excess chloroform was distilled off and crude solid was filtered and dried. The precipitated compound was recrystallized from acetone.

### Refinement

The dibromoethane linkage is disordered over two positions with refined occupancies of 0.834 (6):0.166 (6). The C-Br distances were restrained to be equal, and C_sp^2^_-C_sp^3^_ and C_sp^3^_-C_sp^3^_ distances involving the disordered atoms were restrained to 1.50 (1) and 1.54 (1) Å, respectively. The U^ij^ components of disordered atoms were restrained to approximate isotropic behaviour. H atoms were positioned geometrically [C—H = 0.95 or 1.00 Å] and refined using a riding model, with *U*_iso_(H) = 1.2*U*_eq_(C).

### Crystal data

**Table:**

C_15_H_9_Br_2_Cl_2_FO	*F*_000_ = 1760
*M**_r_* = 454.94	*D*_x_ = 1.957 Mg m^−^^3^
Orthorhombic, *P**b**c**a*	Mo *K*α radiation λ = 0.71073 Å
Hall symbol: -P 2ac 2ab	Cell parameters from 4782 reflections
*a* = 7.1232 (1) Å	θ = 2.8–28.1º
*b* = 10.0757 (2) Å	µ = 5.60 mm^−^^1^
*c* = 43.0262 (7) Å	*T* = 100 (2) K
*V* = 3088.04 (9) Å^3^	Block, colourless
*Z* = 8	0.40 × 0.24 × 0.14 mm

### Data collection

**Table:**

Bruker SMART APEXII CCD area-detector diffractometer	5857 independent reflections
Radiation source: fine-focus sealed tube	3681 reflections with *I* > 2σ(*I*)
Monochromator: graphite	*R*_int_ = 0.056
*T* = 100(2) K	θ_max_ = 33.2º
φ and ω scans	θ_min_ = 1.0º
Absorption correction: multi-scan(SADABS; Bruker, 2005)	*h* = −10→8
*T*_min_ = 0.211, *T*_max_ = 0.508	*k* = −15→15
26343 measured reflections	*l* = −52→66

### Refinement

**Table:**

Refinement on *F*^2^	Secondary atom site location: difference Fourier map
Least-squares matrix: full	Hydrogen site location: inferred from neighbouring sites
*R*[*F*^2^ > 2σ(*F*^2^)] = 0.041	H-atom parameters constrained
*wR*(*F*^2^) = 0.089	*w* = 1/[σ^2^(*F*_o_^2^) + (0.0309*P*)^2^ + 2.3603*P*] where *P* = (*F*_o_^2^ + 2*F*_c_^2^)/3
*S* = 0.99	(Δ/σ)_max_ = 0.001
5857 reflections	Δρ_max_ = 0.77 e Å^−^^3^
227 parameters	Δρ_min_ = −0.76 e Å^−^^3^
60 restraints	Extinction correction: none
Primary atom site location: structure-invariant direct methods	

### Special details

**Table:**

Experimental. The data was collected with the Oxford Cyrosystem Cobra low–temperature attachment.
Geometry. All e.s.d.'s (except the e.s.d. in the dihedral angle between two l.s. planes) are estimated using the full covariance matrix. The cell e.s.d.'s are taken into account individually in the estimation of e.s.d.'s in distances, angles and torsion angles; correlations between e.s.d.'s in cell parameters are only used when they are defined by crystal symmetry. An approximate (isotropic) treatment of cell e.s.d.'s is used for estimating e.s.d.'s involving l.s. planes.
Refinement. Refinement of *F*^2^ against ALL reflections. The weighted *R*-factor *wR* and goodness of fit *S* are based on *F*^2^, conventional *R*-factors *R* are based on *F*, with *F* set to zero for negative *F*^2^. The threshold expression of *F*^2^ > σ(*F*^2^) is used only for calculating *R*-factors(gt) *etc*. and is not relevant to the choice of reflections for refinement. *R*-factors based on *F*^2^ are statistically about twice as large as those based on *F*, and *R*- factors based on ALL data will be even larger.

### Fractional atomic coordinates and isotropic or equivalent isotropic displacement parameters (Å^2^)

**Table:**

	*x*	*y*	*z*	*U*_iso_*/*U*_eq_	Occ. (<1)
Cl1	0.58123 (10)	0.31692 (7)	−0.003788 (15)	0.02739 (16)	
Cl2	0.62659 (10)	−0.10513 (7)	0.070833 (16)	0.02701 (16)	
F1	0.3512 (3)	0.42768 (15)	0.04559 (4)	0.0314 (4)	
O1	0.3888 (3)	−0.05963 (18)	0.12256 (4)	0.0211 (4)	
C1	0.0038 (4)	0.0604 (3)	0.21381 (6)	0.0229 (6)	
H1	0.0475	−0.0286	0.2151	0.028*	
C2	−0.0915 (4)	0.1152 (3)	0.23859 (6)	0.0243 (6)	
H2	−0.1147	0.0635	0.2567	0.029*	
C3	−0.1533 (4)	0.2449 (3)	0.23723 (7)	0.0273 (6)	
H3	−0.2197	0.2822	0.2543	0.033*	
C4	−0.1181 (4)	0.3209 (3)	0.21087 (7)	0.0260 (6)	
H4	−0.1581	0.4107	0.2100	0.031*	
C5	−0.0250 (5)	0.2651 (3)	0.18597 (7)	0.0290 (7)	
H5	−0.0025	0.3166	0.1679	0.035*	
C6	0.0365 (4)	0.1337 (3)	0.18715 (6)	0.0265 (6)	
C9	0.3635 (4)	0.0567 (3)	0.11688 (6)	0.0189 (5)	
C10	0.4255 (4)	0.1189 (2)	0.08738 (6)	0.0176 (5)	
C11	0.3646 (4)	0.2472 (3)	0.07982 (6)	0.0222 (6)	
H11	0.2883	0.2949	0.0941	0.027*	
C12	0.4138 (4)	0.3047 (3)	0.05211 (6)	0.0216 (6)	
C13	0.5258 (4)	0.2405 (3)	0.03074 (6)	0.0196 (5)	
C14	0.5896 (4)	0.1134 (3)	0.03752 (6)	0.0203 (5)	
H14	0.6673	0.0677	0.0231	0.024*	
C15	0.5397 (4)	0.0535 (2)	0.06529 (6)	0.0185 (5)	
Br1	0.4779 (3)	0.1813 (3)	0.17334 (5)	0.0279 (4)	0.834 (6)
Br2	−0.06956 (14)	0.02708 (17)	0.12693 (3)	0.0296 (2)	0.834 (6)
C7	0.1226 (5)	0.0653 (3)	0.15969 (7)	0.0194 (8)	0.834 (6)
H7	0.1772	−0.0209	0.1668	0.023*	0.834 (6)
C8	0.2737 (5)	0.1420 (4)	0.14272 (7)	0.0190 (8)	0.834 (6)
H8	0.2216	0.2261	0.1339	0.023*	0.834 (6)
Br1A	−0.0440 (7)	0.0638 (6)	0.11951 (14)	0.0265 (8)	0.166 (6)
Br2A	0.4743 (16)	0.1862 (16)	0.1704 (2)	0.0222 (16)	0.166 (6)
C7A	0.2193 (14)	0.0999 (16)	0.1700 (3)	0.028 (5)	0.166 (6)
H7A	0.2405	0.0025	0.1728	0.033*	0.166 (6)
C8A	0.2088 (11)	0.1248 (14)	0.1348 (3)	0.015 (4)	0.166 (6)
H8A	0.2190	0.2225	0.1311	0.018*	0.166 (6)

### Atomic displacement parameters (Å^2^)

**Table:**

	*U*^11^	*U*^22^	*U*^33^	*U*^12^	*U*^13^	*U*^23^
Cl1	0.0264 (4)	0.0359 (4)	0.0198 (3)	0.0014 (3)	0.0055 (3)	0.0042 (3)
Cl2	0.0290 (4)	0.0221 (3)	0.0300 (3)	0.0083 (3)	0.0061 (3)	−0.0018 (3)
F1	0.0442 (11)	0.0200 (8)	0.0302 (9)	0.0078 (8)	0.0112 (8)	0.0069 (7)
O1	0.0246 (11)	0.0173 (9)	0.0214 (9)	0.0019 (8)	−0.0011 (8)	−0.0018 (7)
C1	0.0237 (15)	0.0222 (13)	0.0229 (13)	−0.0004 (12)	0.0020 (11)	0.0000 (10)
C2	0.0224 (15)	0.0293 (15)	0.0212 (13)	−0.0056 (12)	0.0026 (11)	0.0010 (11)
C3	0.0224 (16)	0.0323 (16)	0.0271 (15)	−0.0049 (13)	0.0061 (12)	−0.0067 (12)
C4	0.0217 (15)	0.0226 (14)	0.0338 (15)	0.0022 (12)	0.0043 (12)	−0.0038 (12)
C5	0.0381 (19)	0.0219 (14)	0.0270 (15)	0.0049 (13)	0.0102 (13)	0.0045 (11)
C6	0.0322 (18)	0.0248 (14)	0.0226 (13)	0.0052 (13)	0.0082 (12)	0.0013 (11)
C9	0.0190 (14)	0.0173 (12)	0.0203 (13)	−0.0010 (11)	0.0019 (10)	−0.0016 (10)
C10	0.0183 (13)	0.0148 (12)	0.0198 (12)	−0.0012 (10)	0.0021 (10)	−0.0022 (9)
C11	0.0250 (15)	0.0176 (13)	0.0239 (13)	0.0009 (12)	0.0051 (11)	−0.0021 (10)
C12	0.0237 (14)	0.0165 (12)	0.0245 (13)	−0.0004 (11)	0.0011 (11)	0.0019 (10)
C13	0.0182 (14)	0.0249 (14)	0.0158 (12)	−0.0051 (11)	0.0010 (10)	−0.0012 (10)
C14	0.0171 (13)	0.0245 (14)	0.0192 (12)	0.0008 (11)	0.0025 (10)	−0.0054 (10)
C15	0.0144 (13)	0.0165 (12)	0.0244 (13)	−0.0008 (10)	−0.0013 (10)	−0.0034 (10)
Br1	0.0181 (5)	0.0296 (5)	0.0359 (9)	−0.0023 (4)	−0.0012 (5)	−0.0105 (7)
Br2	0.0212 (3)	0.0379 (5)	0.0296 (4)	−0.0013 (3)	−0.0041 (3)	−0.0111 (3)
C7	0.0208 (18)	0.0182 (15)	0.0191 (15)	0.0007 (13)	−0.0027 (13)	−0.0016 (12)
C8	0.022 (2)	0.0175 (16)	0.0173 (17)	−0.0011 (15)	−0.0004 (15)	−0.0024 (12)
Br1A	0.0242 (14)	0.0302 (17)	0.0252 (16)	−0.0071 (12)	−0.0074 (11)	0.0019 (12)
Br2A	0.030 (3)	0.031 (3)	0.0061 (13)	−0.008 (2)	0.0041 (14)	−0.0099 (13)
C7A	0.027 (8)	0.021 (7)	0.035 (8)	−0.010 (6)	−0.010 (6)	0.006 (6)
C8A	0.015 (7)	0.014 (7)	0.016 (7)	−0.005 (6)	−0.007 (5)	0.003 (5)

### Geometric parameters (Å, °)

**Table:**

Cl1—C13	1.719 (3)	C9—C8	1.544 (4)
Cl2—C15	1.731 (3)	C10—C11	1.402 (4)
F1—C12	1.346 (3)	C10—C15	1.414 (4)
O1—C9	1.211 (3)	C11—C12	1.371 (4)
C1—C2	1.380 (4)	C11—H11	0.95
C1—C6	1.384 (4)	C12—C13	1.379 (4)
C1—H1	0.95	C13—C14	1.390 (4)
C2—C3	1.380 (4)	C14—C15	1.385 (4)
C2—H2	0.95	C14—H14	0.95
C3—C4	1.391 (4)	Br1—C8	2.002 (4)
C3—H3	0.95	Br2—C7	2.002 (3)
C4—C5	1.380 (4)	C7—C8	1.513 (4)
C4—H4	0.95	C7—H7	1.00
C5—C6	1.395 (4)	C8—H8	1.00
C5—H5	0.95	Br1A—C8A	2.013 (8)
C6—C7	1.499 (4)	Br2A—C7A	2.014 (8)
C6—C7A	1.536 (9)	C7A—C8A	1.535 (9)
C9—C10	1.483 (4)	C7A—H7A	1.00
C9—C8A	1.509 (9)	C8A—H8A	1.00
			
C2—C1—C6	120.6 (3)	C12—C13—C14	118.8 (2)
C2—C1—H1	119.7	C12—C13—Cl1	119.9 (2)
C6—C1—H1	119.7	C14—C13—Cl1	121.3 (2)
C1—C2—C3	120.2 (3)	C15—C14—C13	119.9 (2)
C1—C2—H2	119.9	C15—C14—H14	120.0
C3—C2—H2	119.9	C13—C14—H14	120.0
C2—C3—C4	119.9 (3)	C14—C15—C10	121.6 (2)
C2—C3—H3	120.1	C14—C15—Cl2	115.5 (2)
C4—C3—H3	120.1	C10—C15—Cl2	122.9 (2)
C5—C4—C3	119.7 (3)	C6—C7—C8	115.9 (3)
C5—C4—H4	120.2	C6—C7—Br2	111.3 (2)
C3—C4—H4	120.2	C8—C7—Br2	104.2 (2)
C4—C5—C6	120.6 (3)	C6—C7—H7	108.4
C4—C5—H5	119.7	C8—C7—H7	108.4
C6—C5—H5	119.7	Br2—C7—H7	108.4
C1—C6—C5	118.9 (3)	C7—C8—C9	110.9 (3)
C1—C6—C7	118.5 (3)	C7—C8—Br1	107.5 (2)
C5—C6—C7	122.4 (3)	C9—C8—Br1	106.5 (2)
C1—C6—C7A	115.1 (6)	C7—C8—H8	110.6
C5—C6—C7A	117.3 (6)	C9—C8—H8	110.6
O1—C9—C10	122.5 (2)	Br1—C8—H8	110.6
O1—C9—C8A	116.5 (6)	C8A—C7A—C6	113.4 (8)
C10—C9—C8A	117.6 (6)	C8A—C7A—Br2A	89.1 (7)
O1—C9—C8	117.1 (2)	C6—C7A—Br2A	131.6 (9)
C10—C9—C8	120.3 (2)	C8A—C7A—H7A	106.7
C11—C10—C15	116.9 (2)	C6—C7A—H7A	106.7
C11—C10—C9	119.7 (2)	Br2A—C7A—H7A	106.7
C15—C10—C9	123.4 (2)	C9—C8A—C7A	113.2 (8)
C12—C11—C10	120.8 (2)	C9—C8A—Br1A	110.3 (6)
C12—C11—H11	119.6	C7A—C8A—Br1A	108.4 (8)
C10—C11—H11	119.6	C9—C8A—H8A	108.3
F1—C12—C11	119.0 (2)	C7A—C8A—H8A	108.3
F1—C12—C13	119.0 (2)	Br1A—C8A—H8A	108.3
C11—C12—C13	122.0 (2)		
			
C6—C1—C2—C3	−0.9 (4)	C1—C6—C7—C8	−137.6 (3)
C1—C2—C3—C4	−0.5 (4)	C5—C6—C7—C8	47.5 (4)
C2—C3—C4—C5	1.3 (4)	C7A—C6—C7—C8	−44.3 (10)
C3—C4—C5—C6	−0.9 (5)	C1—C6—C7—Br2	103.6 (3)
C2—C1—C6—C5	1.4 (5)	C5—C6—C7—Br2	−71.2 (4)
C2—C1—C6—C7	−173.7 (3)	C7A—C6—C7—Br2	−163.1 (11)
C2—C1—C6—C7A	148.2 (6)	C6—C7—C8—C9	174.9 (3)
C4—C5—C6—C1	−0.5 (5)	Br2—C7—C8—C9	−62.5 (3)
C4—C5—C6—C7	174.4 (3)	C6—C7—C8—Br1	58.9 (3)
C4—C5—C6—C7A	−146.6 (6)	Br2—C7—C8—Br1	−178.49 (17)
O1—C9—C10—C11	169.1 (3)	O1—C9—C8—C7	−38.7 (4)
C8A—C9—C10—C11	10.9 (5)	C10—C9—C8—C7	145.0 (3)
C8—C9—C10—C11	−14.8 (4)	C8A—C9—C8—C7	55.5 (16)
O1—C9—C10—C15	−8.5 (4)	O1—C9—C8—Br1	77.9 (3)
C8A—C9—C10—C15	−166.7 (4)	C10—C9—C8—Br1	−98.3 (3)
C8—C9—C10—C15	167.6 (3)	C8A—C9—C8—Br1	172.1 (18)
C15—C10—C11—C12	0.4 (4)	C1—C6—C7A—C8A	151.0 (9)
C9—C10—C11—C12	−177.3 (3)	C5—C6—C7A—C8A	−61.6 (12)
C10—C11—C12—F1	179.5 (2)	C7—C6—C7A—C8A	46.7 (9)
C10—C11—C12—C13	−0.7 (4)	C1—C6—C7A—Br2A	−97.9 (12)
F1—C12—C13—C14	−179.8 (2)	C5—C6—C7A—Br2A	49.5 (13)
C11—C12—C13—C14	0.3 (4)	C7—C6—C7A—Br2A	157.8 (19)
F1—C12—C13—Cl1	−0.7 (4)	O1—C9—C8A—C7A	49.1 (10)
C11—C12—C13—Cl1	179.4 (2)	C10—C9—C8A—C7A	−151.4 (8)
C12—C13—C14—C15	0.3 (4)	C8—C9—C8A—C7A	−48.3 (12)
Cl1—C13—C14—C15	−178.8 (2)	O1—C9—C8A—Br1A	−72.6 (8)
C13—C14—C15—C10	−0.5 (4)	C10—C9—C8A—Br1A	86.9 (8)
C13—C14—C15—Cl2	179.5 (2)	C8—C9—C8A—Br1A	−170 (2)
C11—C10—C15—C14	0.2 (4)	C6—C7A—C8A—C9	−163.6 (8)
C9—C10—C15—C14	177.8 (3)	Br2A—C7A—C8A—C9	60.7 (10)
C11—C10—C15—Cl2	−179.9 (2)	C6—C7A—C8A—Br1A	−40.8 (13)
C9—C10—C15—Cl2	−2.2 (4)	Br2A—C7A—C8A—Br1A	−176.6 (8)

### Hydrogen-bond geometry (Å, °)

**Table:**

*D*—H···*A*	*D*—H	H···*A*	*D*···*A*	*D*—H···*A*
C5—H5···O1^i^	0.95	2.45	3.392 (4)	170
C8—H8···O1^i^	1.00	2.35	3.336 (4)	169
C11—H11···O1^i^	0.95	2.29	3.229 (3)	170
C3—H3···Cg1^ii^	0.95	2.96	3.652 (3)	131

Symmetry codes: (i) −*x*+1/2, *y*+1/2, *z*; (ii) *x*−3/2, *y*, −*z*−1/2.

## References

[bb1] Agrinskaya, N. V., Lukoshkin, V. A., Kudryavtsev, V. V., Nosova, G. I., Solovskaya, N. A. & Yakimanski, A. V. (1999). *Phys. Solid State*, **41**, 1914–1917.

[bb2] Allen, F. H., Kennard, O., Watson, D. G., Brammer, L., Orpen, A. G. & Taylor, R. (1987). *J. Chem. Soc. Perkin Trans. 2*, pp. S1–S19.

[bb3] Bruker (2005). *APEX2*, *SAINT* and *SADABS* Bruker AXS Inc., Madison, Wisconsin, USA.

[bb4] John Kiran, A., Mithun, A., Shivarama Holla, B., Shashikala, H. D., Umesh, G. & Chandrasekharan, K. (2007). *Opt. Commun.* **269**, 235–240.

[bb5] Patil, P. S., Dharmaprakash, S. M., Fun, H.–K. & Karthikeyan, M. S. (2006). *J. Cryst. Growth*, **297**, 111–116.

[bb6] Sheldrick, G. M. (2008). *Acta Cryst.* A**64**, 112–122. 10.1107/S0108767307043930 18156677

[bb7] Shivarama Holla, B., Sooryanarayana Rao, B., Sarojini, B. K., Akberali, P. M. & Suchetha Kumari, N. (2006). *Eur. J. Med. Chem.* **41**, 657–663. 10.1016/j.ejmech.2006.02.001 16616396

[bb8] Spek, A. L. (2003). *J. Appl. Cryst.* **36**, 7–13.

